# TBC1D2 Promotes Ovarian Cancer Metastasis *via* Inducing E-Cadherin Degradation

**DOI:** 10.3389/fonc.2022.766077

**Published:** 2022-04-27

**Authors:** Jiming Tian, Xiaolei Liang, Dalin Wang, Jinglin Tian, Haiping Liang, Ting Lei, Zeyu Yan, Dan Wu, Xiaoli Liu, Shujuan Liu, Yongxiu Yang

**Affiliations:** ^1^ The First Clinical Medical College of Lanzhou University, Lanzhou, China; ^2^ Department of Obstetrics and Gynecology, Key Laboratory for Gynecologic Oncology Gansu Province, The First Hospital of Lanzhou University, Lanzhou, China; ^3^ State Key Laboratory of Cancer Biology and Department of Physiology and Pathophysiology, Fourth Military Medical University, Xi’an, China; ^4^ Department of Gynecology and Obstetrics, Xijing Hospital, Fourth Military Medical University, Xi’an, China

**Keywords:** ovarian cancer, metastasis, TBC family, TBC1D2, E-cadherin

## Abstract

**Background:**

Ovarian cancer (OC) is the most lethal gynecological malignancy worldwide. Increasing evidence indicates that TBC domain family is implicated in various cellular events contributing to initiation and development of different cancers, including OC. However, the role of TBC1D2, a crucial member of TBC domain family, remains unclear in OC.

**Methods:**

IHC and qRT-PCR were employed to determine TBC1D2 expression in OC tissues and cells. *In vitro* and *in vivo* assays involving proliferation, migration, invasion were performed to explore the role of TBC1D2 in OC development. The underlying mechanism by which TBC1D2 promotes OC metastasis were elucidated using bioinformatics analysis, western blotting and co-immunoprecipitation.

**Results:**

Upregulation of TBC1D2 was found in OC and was associated with a poor prognosis. Meanwhile, TBC1D2 promoted OC cell proliferation, migration, and invasion *in vitro* and facilitated tumor growth and metastasis *in vivo*. Moreover, TBC1D2 contributed to OC cell invasion by E-cadherin degradation *via* disassembling Rac1-IQGAP1 complex. In addition, miR-373-3p was screened out and identified to inhibit OVCAR3 invasion *via* negative regulation of TBC1D2.

**Conclusion:**

Our findings indicated that TBC1D2 is overexpressed in OC and contributes to tumor metastasis *via* E-cadherin degradation. This study suggests that TBC1D2 may be an underlying therapeutic target for OC.

## Introduction

Ovarian cancer (OC) is one of the three most common gynecological malignancies with the highest mortality rate among all gynecological malignancies (1). In 2020, an estimated 313,959 new cases of ovarian cancer occurred, accompanying 207,252 deaths worldwide (2). Due to the lack of specific early symptoms and effective screening strategies, up to 70% of high-grade serous ovarian cancer patients are in advanced stage along with metastasis, with a 5-year overall survival (OS) rate less than 30% (1, 3). Although a great deal of work has been conducted on early detection and diagnosis of ovarian cancer, numerous trials have failed to identify effective approaches and biomarkers (4). From this perspective, it is imperative to focus on the underlying mechanisms and targeted molecular agents of ovarian cancer.

TBC/RabGAPs (Tre2–Bub2–Cdc16 domain-containing RAB-specific GAPs) family contains a highly conserved TBC domain that inactivates Rabs by facilitating hydrolysis of Rab-associated guanosine triphosphate (GTP) into guanosine diphosphate (GDP) (5). TBC1D2, a key member of TBC/RabGAPs family, mediates crosstalk between Rac1 activation and Rab7 cycling (6, 7). TBC1D2 also coordinates the function of these two small GTPases during specific biological processes, including scattering, transport, and autophagy (8). Besides, TBC1D2 modulates Rab7 cycling through Rac1 activation to promote E-cadherin degradation in EGF-induced scattering and Arf6-dependent disassembly of junctions (7). Moreover, TBC1D2 regulates endocytic trafficking by inhibiting Rab7, a key regulator of lysosomal function (8). However, the biological functions and potential mechanisms of TBC1D2 in human OC remain unknown.

The prognosis of patients with ovarian cancer are tightly linked with tumor metastasis, and metastatic dissemination accounts for a vast percentage of mortality (9). The adherens junctions are the most vital cell-cell adhesion parts which are responsible for maintaining a coherent primary tumor mass. And disruption or disintegration of adherens junctions, which are composed of E-cadherin/β-catenin/α-catenin complexes, contributes to the tumor dissociation, dissemination and metastasis (10). E-cadherin, a member of the cadherin family, plays a major role in maintaining intercellular adhesion and controls metastatic progression (11). What’s more, an inverse correlation was demonstrated between E-cadherin expression and tumor cell invasion and motility and similarly with metastatic disease in cancer patients (12).

Herein, to elucidate the vital role of TBC1D2 in ovarian carcinogenesis and development, we performed bioinformatic analysis and a series of functional experiments *in vitro* and *in vivo*. Our study revealed that TBC1D2 is overexpressed in ovarian cancer and is associated with a poor prognosis. Moreover, TBC1D2 promotes migration and invasion of OC cells *via* E-cadherin degradation. Our findings indicate that TBC1D2 may be a promising therapeutic target for OC therapy.

## Material and Methods

### Cell Culture and Tissue Collection

The epithelial ovarian cancer cells lines, including OVCAR3, A2780 and SKOV3 were obtained from the National Collection of Authenticated Cell Cultures (Shanghai, China). OVCAR3 and A2780 cells were cultured in RPMI-1640 medium, whereas SKOV3 cells were cultured in DMEM (Thermo Fisher Scientific, Waltham, MA, USA). They were supplemented with 10% fetal bovine serum (Sangon Biotech, Shanghai, China) and 1% penicillin-streptomycin (Solarbio, Beijing, China) at 37°C in 5% CO2. All OC cell lines were authenticated using short tandem repeat DNA testing at the Center for DNA Typing of the Fourth Military Medical University (FMMU, Xi’an, China).

OC tissue samples were collected from 100 patients who underwent surgery without neoadjuvant chemotherapy at the Department of Obstetrics and Gynecology of the First Hospital of Lanzhou University (Lanzhou, China) from 2011 to 2020. The clinical characteristics of OC patients are listed in **Supplementary Table S1**. All participants provided written informed consent. This study was approved by the Ethics Committee of the First Hospital of Lanzhou University.

### Collection of Public Datasets and Bioinformatics Analysis

TCGA-OV dataset was obtained from the TCGA data portal (https://portal.gdc.cancer.gov/) in March 2021, containing transcriptome profiling data on 374 OC samples with clinical information, including survival time and survival status. Clinical characteristics of patients from TCGA-OV cohort are summarized in **Supplementary Table S2**. Three mRNA expression datasets of OC tissues [GSE66957, GSE12470 (13) and GSE40595 (14)] were downloaded from the Gene Expression Omnibus database (**Supplementary Table S3**). Gene Expression Profiling Interactive Analysis (GEPIA2, http://GEPIA2.cancer-pku.cn) was used to analyze TBC1D2 mRNA expression and patient survival of TCGA-OV dataset.

### Plasmid Construction and Transfection

To overexpress TBC1D2 in ovarian cancer cells, its coding sequence was amplified from cDNA derived from A2780 cells using primers containing BamHI and EcoRV restriction sites and then cloned into pcDNA3.1 expression vector (Thermo Fisher Scientific, Waltham, MA, USA). To generate small hairpin RNA expression vectors, the small hairpin RNA containing specific sequences targeting the human TBC1D2 mRNA sequence was cloned into pGPU/GFP/Neo vector (Genepharma, Shanghai, China). All Small interfering RNAs (siRNAs) were constructed by GenePharma (Shanghai, China) and their sequences are listed in **Supplementary Table S4**. The cells were plated in 6-well plates at approximately 70% confluence 24 h before transfection using Lipofectamine 2000 (Thermo Fisher Scientific, Waltham, MA, USA) according to manufacturer’s protocol. Briefly, 4.5 μg plasmid or 8 μL siRNA treatments were mixed with 500 μL Opti-MEM containing 8 μL Lipofectamine 2000 for 6 h. Western blotting or functional studies were performed 72 h after transfection. Cell lines stably expressing shTBC1D2 or TBC1D2 in OVCAR3 and A2780 cells were established using G418 sulfate (OVCAR3, 400 μg/mL, A2780, 500 μg/mL for five days; Millipore Sigma, Burlington, MA, USA) treatment following transfection. The primers used are listed in **Supplementary Table S4**.

### Cell Viability Assay

A cell counting kit-8(#C0039, Beyotime, Shanghai, China) was used to perform cell counting. Briefly, cells were seeded into 96-well plates at 2,000 cells/well. After the cells adhered, add 100 μl of medium containing 10% CCK-8 reagent and incubate for 1 hour. The absorbance value was detected at 450 nm using a microplate reader.

### Cell Proliferation Assay

5-Ethynyl-2’-deoxyuridine (EdU) incorporation assay was performed according to manufacturer’s protocol (#C10310-1; Ribobio, Guangzhou, China). Briefly, 2×10^4^ cells/well were seeded into 24-well plates. One day after seeding, each well received 200 μL of complete medium with 50 μM EdU. OC cells were incubated for 1 h at 37°C. After fixation with 4% paraformaldehyde, the cells were stained with Hoechst and Apollo reaction cocktail. A fluorescence microscope was used to capture images. Following that, the ratio of EdU-positive cells to total cells within each microscopic was calculated. The experiment was performed in triplicate.

### Apoptosis Assay

Following transfection with siRNA or expression vectors, ovarian cancer cells were cultured for two days. An Annexin V-FITC apoptosis detection kit (#BB-4101-3; Bestbio, Nanjing, China) was used according to manufacturer’s instructions. Fluorescence was detected using a flow cytometer (Beckman Coulter, CA, USA). The experiment was performed in triplicate.

### Wound Healing Assay

The cells were plated in 6-well plates. When the cells reached confluence, we gently drew a vertical line with a plastic yellow pipette tip to create a mechanical wound. After washing with phosphate-buffered saline, the cells were incubated in a serum-free medium at 37°C. The healing conditions were photographed under a light microscope at 0 and 24 h. The scratch area was measured using ImageJ software. Relative migration rate = 24 h healing area/0 h wound area.

### Transwell Migration and Invasion Assay

The cell invasion assay was performed using a 24-well transwell chamber (BD Biosciences, CA, USA). Matrigel was added to the upper chamber to mimic the normal extracellular matrix and incubated overnight. Subsequently, each upper chamber was supplemented with 2×10^4^/100 μL OC cells. Each lower chamber contained 500 μL of DMEM and was supplemented with 20% FBS. After incubation for 48 h, the penetrated cells were fixed with 4% paraformaldehyde and stained with crystal violet. Finally, the invasive cells were photographed and counted. All assays were performed in triplicate.

### Animal Experiment

The animal studies were conducted in accordance with National Institutes of Health (NIH) guidelines and approved by the Animal Care Committee of the Fourth Military Medical University (FMMU). For mouse xenograft experiment, 1×10^7^ cells were injected subcutaneously into the flanks of BALB/c nude mice (4-6 weeks old). Tumor growth was evaluated weekly after injection. Tumor length (L) and width (W) were measured using a Vernier caliper, and the tumor volume (V) was calculated according to the formula *V*=*L* × *W* ^2^/2. After 28 days, the mice were euthanized.

For *in vivo* metastasis assays, 1×10^7^ cells were injected intraperitoneally into BALB/c nude mice (female, 4-6 weeks old). Survival status and the production of ascites were observed every week after injection. Five weeks after injection, the nude mice were euthanized, dissected and photographed.

### Functional Enrichment Analysis

Gene ontology (GO) biological process and Kyoto encyclopedia of genes and genomes (KEGG) pathways were explored using genetrail3.0 (http://genetrail.bioinf.uni-sb.de/) (15), a website focused on advanced high-throughput enrichment analysis. The groups were divided according to TBC1D2 expression levels. And GSEA software(version, 4.0.3) was used to examine the distribution of the curated gene sets from the Broad Institute’s MsigDB (http://www.broadinstitute.org/GSEA/msigdb/index.jsp) in lists of genes ordered according to TBC1D2 expression (16) (**Supplementary Table S5**).

### RNA Extraction, qRT-PCR and Western Blotting

Total RNA was extracted from ovarian cancer cells using E.Z.N.A.^®^Total RNA Kit I (#R6834; OMEGA Bio-Tek, Inc., GA, USA) according to manufacturers’ instructions, and 500 ng of total RNA was used in each reverse transcription reaction with PrimeScript™ RT reagent Kit with gDNA Eraser (Perfect Real Time) (#RR047A, TaKaRa, Beijing, China), according to manufacturers’ protocol. For western blotting, the cells were lysed in RIPA lysis buffer (#P0013B, Beyotime, Shanghai, China) supplemented with protease inhibitor cocktail (Invitrogen) for 30 min and then centrifuged at 12,000 × g for 10 min at 4°C. The protein supernatant was then boiled in 5×protein loading buffer for 5 min at 100°C, separated by SDS-PAGE, transferred onto PVDF membranes, and blocked in 5% skim milk for 1 h at room temperature. Primary antibodies (**Supplementary Table S6**) were incubated overnight at 4°C, and appropriate secondary antibodies were incubated for 1 h at room temperature. Immunoreactive bands were visualized using a ChemiScope 6000 Exp Chemiluminescence Imaging System (Clinx, Shanghai, China) after development with an enhanced chemiluminescence detection kit (Millipore, Darmstadt, Germany).

### Immunohistochemical (IHC) Staining and Analysis

Immunohistochemical staining was performed using an IHC detection kit (Invitrogen, USA). According to manufacturers’ instructions, the antigen was restored by treating with boiling citrate buffer (pH=6.0) under pressure. After that, sections were incubated with primary antibodies at 4°C overnight. The color was developed using 3, 5-diaminobenzidine (DAB) substrate followed by hematoxylin counterstaining. The intensity (0, none; 1, faint yellow; 2, yellow; 3, brown) and proportion of positive cells (0, 0-9%; 1, 10-25%; 2, 26-50%; 3, 51-75%; and 4, 76-100%) were determined in five random microscopic visual sights per slide by two independent pathologists blinded to the clinical data. IHC scoring (0–12) was performed by multiplying the percentage of positively stained cells by the intensity. The median value of IHC score was chosen as the cut-off point for classifying low and high expression.

### Co-Immunoprecipitation Assays

The Pierce Classic Magnetic IP/Co-IP Kit (Cat. No. 88805, Thermo Fisher Scientific, MA) was used for IP. Briefly, lyse the A2780 cells with treatment as indicated (around 1×10^7^ cells) in 400 μL IP buffer (supplemented with 1×proteinase inhibitor cocktail and 1mM PMSF) by incubating on ice for 30 min, and centrifugate at 10,000g for 10 minutes at 4°C to collect protein supernatant. The protein sample was immunoprecipitated with 5-10 µg of antibody overnight at 4°C. Subsequently, 25 µL of fully suspended protein A/G magnetic beads were added and incubated at room temperature for 1.5 h. The complexes that bound to the protein A/G conjugate were washed and subjected to western blotting.

### Luciferase Report Assay

To generate the luciferase reporter plasmid, PCR amplified 3’UTR regions of TBC1D2 containing putative binding sites for miR-373-3p were cloned downstream of firefly luciferase gene of pmirGLO Dual-Luciferase miRNA Target Expression Vector (Promega, WI, USA). The mutant luciferase reporter constructs of TBC1D2 3’UTR were generated using a QuikChange II XL Site-Directed Mutagenesis Kit (#200521, Agilent Technologies, Beijing, China). The PmirGLO empty vector was used as a normalization control. The plasmids described above were co-transfected with miR-373-3p mimic or mimic control following previous transfection procedure. The cells were lysed and analyzed using a Dual-Luciferase Reporter Assay Kit (Promega Corporation, Madison, WI, USA), according to manufacturer’s instructions. The firefly luciferase activity in each group was normalized to Renilla luciferase activity. The relative light units were measured using a microplate reader.

### Statistical Analysis

All experiments were performed independently, at least in triplicate. GraphPad Prism 8.0 software (San Diego, CA, USA) was used for statistical analysis. All data are presented as mean ± standard error of mean (SEM) values from triplicate experiments. Statistical significance was set at P < 0.05. Differences between the two groups were analyzed using an independent samples t-test.

## Results

### TBC1D2 Is Upregulated in Ovarian Cancer and High Expression Level of TBC1D2 Is Associated With Poor Prognosis

**Figure 1:**
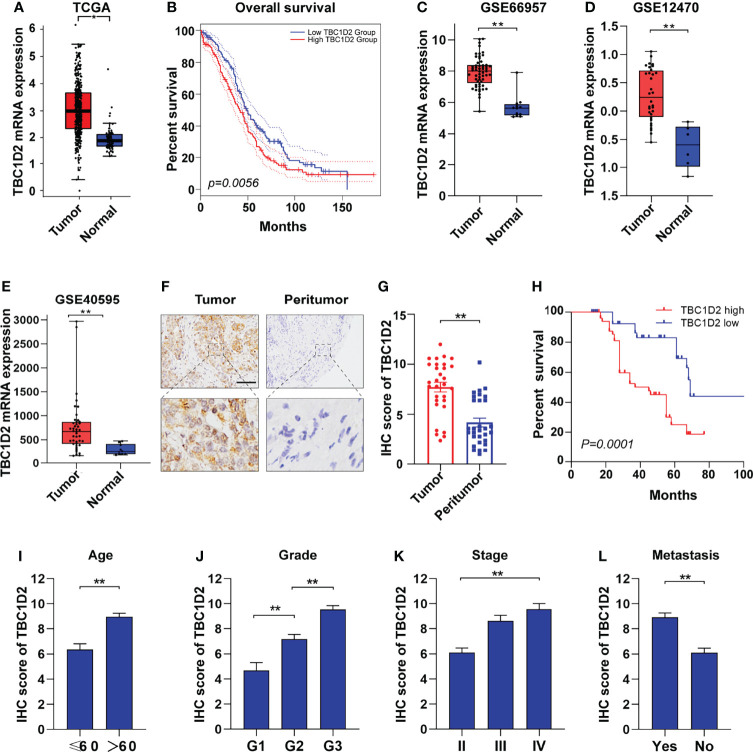
TBC1D2 is upregulated in ovarian cancer and high expression level of TBC1D2 is associated with poor prognosis. **(A)** Expression of TBC1D2 in OC and normal tissues according to GEPIA 2 website. OC, ovarian cancer. **(B)** Overall survival rates of OC patients with high or low TBC1D2 expression level were evaluated by Kaplan–Meier plot and log-rank test according to GEPIA 2 website. **(C–E)** Expression of TBC1D2 in OC and normal tissues in GSE66957, GSE12470 and GSE40595 datasets. **(F)** Representative immunohistochemical staining images of TBC1D2 expression in human OC and peritumor tissues (n = 100). bar, 100μm. **(G)** IHC scores of TBC1D2 in OC tumor and peritumor tissues were evaluated in our cohort. **(H)** Overall survival rates of OC patients with high or low TBC1D2 expression level were evaluated by Kaplan–Meier plot and log-rank test. **(I–L)** IHC scores of TBC1D2 according to age, grade, stage and metastasis were evaluated in our cohort. Two-tailed Student’s t tests were used to determine the significance of differences between two groups; Log-rank tests were used to compare the overall survival between two groups; data are represented as mean ± SEM. *P < 0.05, **P < 0.01.

To explore the role of TBC1D2 in ovarian cancer, we analyzed the mRNA expression in tumors from 426 OC samples and relevant normal tissues from 88 samples according to GEPIA 2 website. Much more TBC1D2 mRNA expression was found in tumor tissues compared with that in normal tissues (**Figure 1A**). Kaplan–Meier curves for overall survival of OC patients with different mRNA expression levels of TBC1D2 were plotted. The results revealed that OC patients with higher TBC1D2 mRNA expression (median as cutoff) lived with poorer prognosis (p<0.01; **Figure 1B**). We then analyzed mRNA expression of TBC1D2 using Gene Expression Omnibus database (GSE66957, GSE12470, and GSE40595). As we expected, the findings were consistent with above results based on GEPIA 2 (**Figures 1C–E**). Furthermore, immunohistochemistry was utilized to assess the protein expression level of TBC1D2 in OC tissues and peritumor tissues from 100 OC patients, and the result was in accordance with the findings above involving mRNA level of TBC1D2. (**Figures 1F, G** and **Supplementary Table S7**). Besides, we also found that patients with higher TBC1D2 expression (median as cutoff) had a poorer prognosis (**Figure 1H**). Additionally, TBC1D2 was also significantly increased in OC patients with old age, high grade, advanced stage, and metastasis (**Figures 1I–L**). These results suggested that TBC1D2 plays a vital role in OC development.

### TBC1D2 Promotes Proliferation of OC Cells *In Vitro* and Tumor Growth *In Vivo*

**Figure 2:**
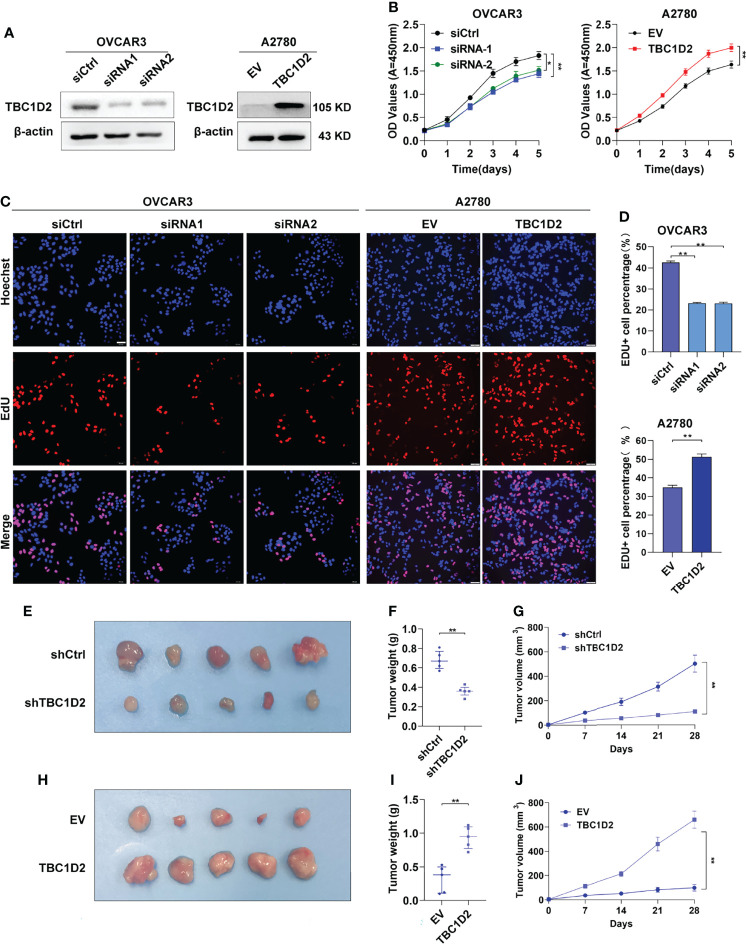
TBC1D2 prompts the proliferation of ovarian cancer cells *in vitro* and tumor growth *in vivo*. **(A)** Western blotting images of TBC1D2 in OVCAR3 and A2780 cells treated as indicated. siRNA1 or siRNA2, siRNA against TBC1D2; siCtrl, control siRNA; EV, empty vector; TBC1D2, overexpression vector encoding TBC1D2. **(B)** Growth curves of ovarian cancer cells treated as indicated. **(C–D)** EdU incorporation assays were performed to assess the proliferation ability of OVCAR3 and A2780 cells treated as indicated. Scale bars, 50μm. **(E, H)** Photographs of nude mouse xenografts generated by subcutaneous injection of OVCAR3 cells or A2780 cells treated as indicated; n = 5/group. **(F, I)** Tumor weights were measured and analyzed 28 days after injection. **(G, J)** Tumor volume curves of subcutaneous xenograft models from OVCAR3 cells or A2780 cells treated as indicated. Tumor volume was measured using vernier calipers every 7 days after subcutaneous implantation. Two-tailed Student’s t tests were used to determine the significance of differences between two groups; data are represented as mean ± SEM. *P < 0.05, **P < 0.01.

The mRNA and protein expression levels of TBC1D2 were evaluated in several OC cell lines, and we found that TBC1D2 expressed most in OVCAR3 cells and barely expressed in A2780 cells (**Supplementary Figures S1A, B**). Subsequently, TBC1D2 knockdown and overexpression were performed respectively in OVCAR3 and A2780 cell lines (**Figure 2A**). To assess the effect of TBC1D2 on OC cell growth, CCK8 assays were performed and the results showed that TBC1D2 knockdown significantly decreased OVCAR3 cell viability, while overexpression of TBC1D2 promoted A2780 cell growth (**Figure 2B**). As depicted in **Figures 2C, D**, OVCAR3 cells with TBC1D2 knockdown showed much less EdU incorporation than the control cells, whereas TBC1D2 overexpression exhibited the opposite effect in A2780 cells. In addition, apoptosis assays revealed that OVCAR3 cells interfering with TBC1D2 activity showed a slight decrease in the percentage of apoptotic cells compared with that of the control group, while the opposite result was observed in A2780 cells (**Supplementary Figures S1C, D**). However, these results were not statistically significant. Next, the subcutaneous xenograft study was performed by subcutaneous injection of TBC1D2 knockdown or overexpressing cells using nude mice. Obviously, xenograft tumors grew smaller and slower in mice injected OVCAR3 cells with TBC1D2 knockdown than those in mice injected the control cells, while tumors grew bigger and faster in mice injected A2780 cells overexpressing TBC1D2 than those in the control group (**Figures 2E–J**).

### TBC1D2 Promotes the Migration and Invasion of OC Cells *In Vitro* and Tumor Metastasis *In Vivo*

**Figure 3:**
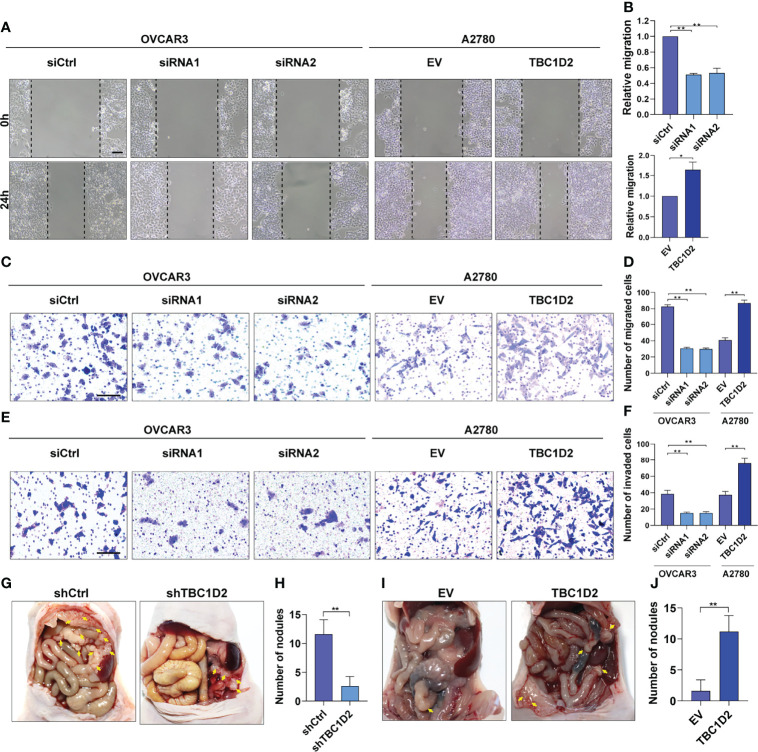
TBC1D2 promotes the migration and invasion of OC cells *in vitro* and tumor metastasis *in vivo*. **(A, B)** Representative images and analysis of wound healing assays in OVCAR3 and A2780 cells treated as indicated. Scale bars, 100μm. **(C, D)** Representative images and analysis of transwell migration assays in OVCAR3 and A2780 cells treated as indicated. Scale bars, 100μm. **(E, F)** Representative images and analysis of transwell-Matrigel invasion assays in OVCAR3 and A2780 cells treated as indicated. siRNA1 or siRNA2, siRNA against TBC1D2; siCtrl, control siRNA; EV, empty vector; TBC1D2, overexpression vector encoding TBC1D2. Scale bars, 100μm. **(G–J)** Representative images of intraperitoneal metastases and number of metastatic nodules in each group were shown and analyzed; n = 5/group. Two-tailed Student’s t tests were used to determine the significance of differences between two groups; data are represented as mean ± SEM. *P < 0.05, **P < 0.01.

To further elucidate the role of TBC1D2 in modulating OC development, wound healing assay and transwell assay involving cell migration and invasion were performed. The results demonstrated that knockdown of TBC1D2 significantly inhibited the ability of cell migration and invasion. In contrast, TBC1D2 overexpression played the opposite role (**Figures 3A–F**). Moreover, an *in vivo* OC metastasis model was constructed in nude mice to assess the effect of TBC1D2 on OC metastasis. As **Figures 3G–J** showed, the ability of OC metastasis and the number of metastatic nodules decreased when TBC1D2 was knocked down in OVCAR3 cells. On the contrary, TBC1D2 overexpression in A2780 cells enhanced the ability of tumor metastasis *in vivo*. Thus, these results imply that TBC1D2 promotes invasion and migration of OC cells and OC metastasis.

### TBC1D2 Promotes Ovarian Cancer Progression by Upregulating RAC1 and IQGAP1 Expression

**Figure 4:**
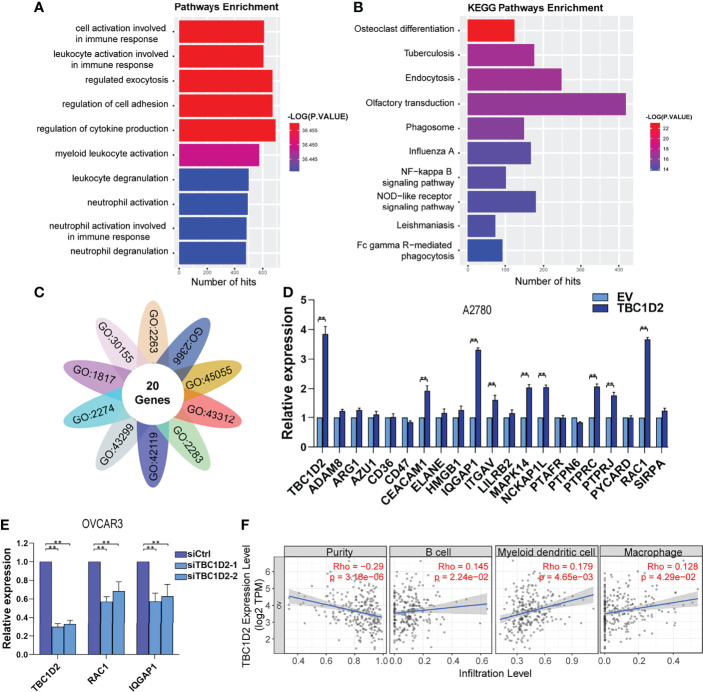
TBC1D2 promotes ovarian cancer progression by upregulating RAC1 and IQGAP1 expression. **(A, B)** GO analysis and KEGG pathway analysis based on the Genetrail3.0 website. **(C)** Venn diagram of 20 overlapping genes based on the significant biological processes shown in **Figure 4A**. **(D)** qRT-PCR analysis of 20 overlapping genes in A2780 cells treated as indicated. **(E)** qRT-PCR analysis of TBC1D2, RAC1 and IQGAP1 in OVCAR3 cells treated as indicated. **(F)** Correlation analysis between TBC1D2 expression and immune cell infiltration in OC. Two-tailed Student’s t tests were used to determine the significance of differences between two groups; data are represented as mean ± SEM. **P < 0.01.

To explore the involved molecules and pathways by which TBC1D2 promotes OC development, the functional enrichment analysis was performed on 374 samples from TCGA. All samples were divided into TBC1D2 high-expression and low-expression groups according to the median of TBC1D2 FPKM. The biological processes and Kyoto Encyclopedia of Genes and Genomes (KEGG) pathways associated with high expression level of TBC1D2 were analyzed through Genetrail3.0 and we found that the results were primarily focused on regulating cell adhesion and immunocyte activation (**Figures 4A, B**). In addition, we also performed other function enrichment based on GSEA software. Similar to the previous results, these pathways were also enriched, including ECM receptor interaction, focal adhesion, B cell receptor signaling pathway, endocytosis, T cell receptor signaling pathway and natural killer cell mediated cytotoxicity (**Supplementary Figure S2A**).

To further determine the significant genes affected by TBC1D2, genes involved in the above biological processes were investigated. Twenty genes were identified to play roles in all ten pathways to promote tumor progression, including ADAM8, ARG1, AZU1, CD36, CD47, CEACAM1, ELANE, HMGB1, IQGAP1, ITGAV, LILRB2, MAPK14, NCKAP1L, PTAFR, PTPN6, PTPRC, PTPRJ, PYCARD, RAC1, and SIRPA (**Figure 4C**). Furthermore, 20 genes were screened using qRT-PCR analysis, and two (RAC1 and IQGAP1) were most significantly upregulated by TBC1D2 in A2780 cells (**Figure 4D** and **Supplementary Figure S2B**). Consistent with the above result, TBC1D2 knockdown significantly decreased RAC1 and IQGAP1 expression (**Figure 4E**), and data from TCGA-OV also supported our findings (**Supplementary Figures S2C, D**). These results indicated that TBC1D2 probably plays a vital biological role in OC progression by upregulating RAC1 and IQGAP1 expression. In addition, to define the specific relationship between TBC1D2 expression and the infiltrative level of different subsets of immune cells in OC microenvironment, Tumor Immune Estimation Resource (TIMER) was employed. As depicted in **Figure 4F**, TBC1D2 expression was positively correlated with several immune cells, including B cells, dendritic cells, and macrophages.

### TBC1D2 Promotes Cell Invasion by Decreasing E-Cadherin *via* Disassembling Rac1-IQGAP1 Complex

RAC1 and IQGAP1 were reported to play key roles in regulating tumor cell adhesion (17). Furthermore, intercellular adherens junctions were stabilized when Rac1 binds to IQGAP1 (18, 19). However, Frasa MA et al. found that TBC1D2 could interact with RAC1 to promote the degradation of E-cadherin and the instability of cell-cell adhesion (7).

**Figure 5:**
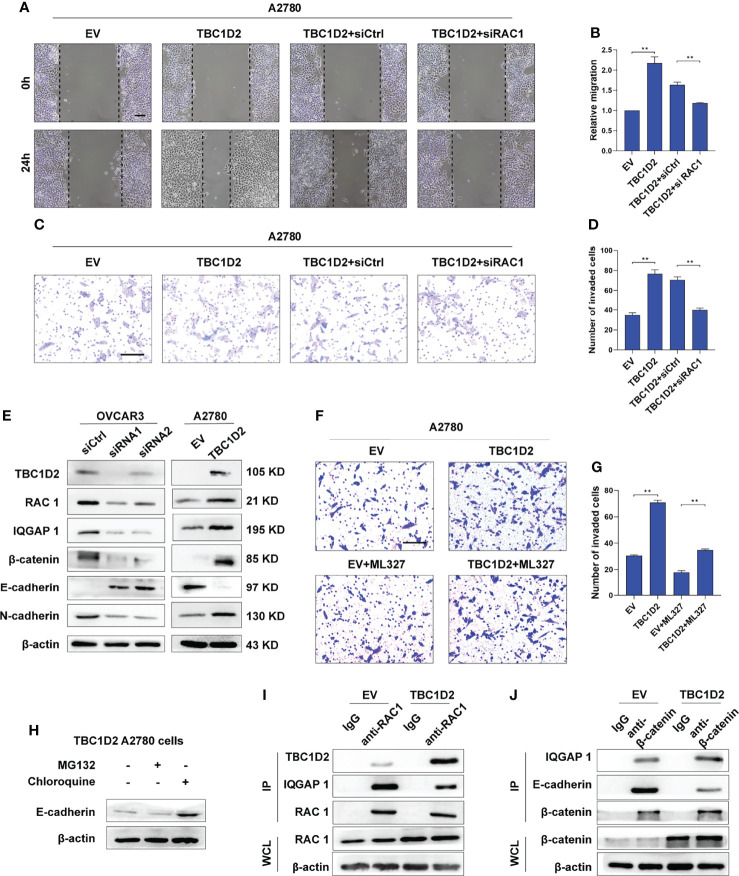
TBC1D2 promotes cell invasion by decreasing E-cadherin *via* disassembling Rac1-IQGAP1 complex. **(A-D)** Representative images and analysis of wound healing assay and transwell invasion assay in A2780 cells treated as indicated. EV, empty vector; TBC1D2, overexpression vector encoding TBC1D2; siCtrl, control siRNA; siRac1, siRNA against Rac1. Scale bars, 100μm **(E)** Western blotting images of TBC1D2, Rac1, IQGAP1, β-catenin, E-cadherin, N-cadherin and β-actin in OVCAR3 and A2780 cells treated as indicated. siRNA1 or siRNA2, siRNA against TBC1D2; siCtrl, control siRNA; EV, empty vector; TBC1D2, overexpression vector encoding TBC1D2. **(F, G)** Representative images and analysis of transwell invasion assay in A2780 cells treated as indicated. EV, empty vector; TBC1D2, overexpression vector encoding TBC1D2. Scale bars, 100μm **(H)** Western blotting images of E-cadherin and β-actin in TBC1D2 overexpressed A2780 cells treated with MG132 or Chloroquine. **(I)** Co-IP of anti-Rac1 with TBC1D2 or IQGAP1 from A2780 cells treated as indicated. Equal amount of immunoprecipitated Rac1 from TBC1D2 overexpressed or control A2780 cells was loaded in WB assay. WCL, whole cell lysates. **(J)** Co-IP of anti-β-catenin with IQGAP1 or E-cadherin from A2780 cells treated as indicated. Equal amount of immunoprecipitated β-catenin from TBC1D2 overexpressed or control A2780 cells was loaded in WB assay. WCL, whole cell lysates. Two-tailed Student’s t tests were used to determine the significance of differences between two groups; data are represented as mean ± SEM. **P < 0.01.

To investigate the specific mechanism by which TBC1D2 promotes ovarian cancer progression, we studied at first if TBC1D2 modulates OC cell migration and invasion through regulating RAC1 and IQGAP1. Therefore, RAC1 was interfered in A2780 cells stably overexpressing TBC1D2 and we found that RAC1 knockdown significantly suppressed migration and invasion of A2780 cells (**Figures 5A–D**). Similar phenomenon of IQGAP1 knockdown was observed compared with Rac1 knockdown (**Supplementary Figures S3A–D**). We then wonder whether TBC1D2 modulates the key molecules involving E-cadherin-mediated intercellular adhesion. Interestingly, the results of western blotting showed that TBC1D2 overexpression significantly decreased E-cadherin expression and increased the expression of Rac1, IQGAP1, β-catenin, and N-cadherin in A2780 cells, and vice versa in OVCAR3 cells (**Figure 5E**). Further, we investigated if TBC1D2 modulated the protein level of E-cadherin *via* RAC1 and IQGAP1 using western blotting. The results showed that interfering with RAC1 was able to weaken the increased IQGAP1 and decreased E-cadherin induced by TBC1D2 overexpression in A2780 cells, but interfering with IQGAP1 just weaken the decreased E-cadherin induced by TBC1D2 overexpression in A2780, which indicated that E-cadherin played a downstream role of Rac1 and IQGAP1 (**Supplementary Figures S3E, F**). Subsequently, ML327, a compound which plays a part in restoring E-cadherin expression, was used to perform a rescue experiment (**Supplementary Figure S3G**). The result of Transwell assay revealed that E-cadherin induced by ML327 (10 μM) significantly impaired the invasion capacity when TBC1D2 was overexpressed in A2780 cells (**Figures 5F, G**). Our findings indicated that TBC1D2 promoted OC cell invasion by RAC1 and IQGAP1 induced reduction of E-cadherin.

Afterwards, to investigate the specific pathway involving E-cadherin downregulation when the cadherin junctions were disassembled, A2780 cells with TBC1D2 overexpression were treated with MG132 were treated with MG132 (a proteasome inhibitor, 10 μM, 12 hours) or chloroquine (a lysosome inhibitor, 100 μM, 12 hours) to suppress the degradation of proteins, and western blotting was used to evaluate the amount of E-cadherin. As demonstrated in **Figure 5H**, cells treated with chloroquine, not MG132, showed increased amounts of E-cadherin. The result indicated that it was lysosomal degradation that accounted for downregulation of E-cadherin after overexpression of TBC1D2. Furthermore, co-immunoprecipitation assays were used to demonstrated the underlying intermolecular interactions in A2780 cells. As shown in **Figures 5I, J**, overexpression of TBC1D2 increased the bindings of TBC1D2 to Rac1, leading to disintegration of Rac1-IQGAP1 complex. Thus, the bindings of free IQGAP1 to β-catenin increased, resulting in dissociation of E-cadherin-mediated intercellular adhesion. Taken together, these results indicates that TBC1D2 promotes cell invasion by lysosomal degradation of E-cadherin *via* disrupting Rac1-IQGAP1 complex.

### miR-373-3p Inhibits Invasion of OVCAR3 Cells *via* Negative Regulation of TBC1D2

**Figure 6:**
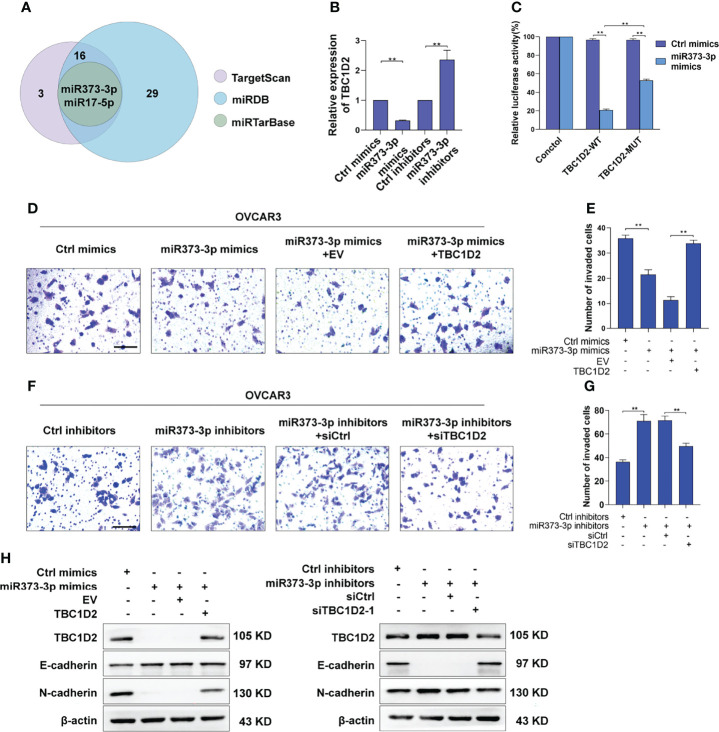
miR-373-3p inhibits invasion of OVCAR3 cells *via* negative regulation of TBC1D2. **(A)** Venn diagram of the putative miRNAs targeting TBC1D2 from miRDB, miRTarBase and TargetScan databases. **(B)** qRT-PCR analysis of TBC1D2 expression in OVCAR3 cells following transfection with miR-373-3p mimics or inhibitors. **(C)** The regulatory capacity of miR-373-3p on TBC1D2 was assessed by luciferase reporter assay in OVCAR3 cells treated as indicated. WT, wild type; MUT, mutation. **(D-G)** Representative images and analysis of transwell invasion assay in OVCAR3 cells treated as indicated. EV, empty vector; TBC1D2, overexpression vector encoding TBC1D2; siCtrl, control siRNA; siTBC1D2-1, siRNA against TBC1D2. Scale bars, 100μm **(H)** Western blotting images of TBC1D2, E-cadherin, N-cadherin and β-actin in OVCAR3 cells treated as indicated. Two-tailed Student’s t tests were used to determine the significance of differences between two groups; data are represented as mean ± SEM. **P < 0.01.

Numerous miRNAs are thought to act as tumor suppressors in various human malignancies (20, 21). To investigate whether miRNAs are involved in modulating TBC1D2 expression, potential miRNAs were predicted using miRDB (22), miRTarBase (23), and TargetScan databases (24). As depicted in **Figure 6A**, two underlying miRNAs (miR-373-3p and miR-17-5p) targeting TBC1D2 were identified. Although miR-17-5p has been previously reported to regulate endocytic trafficking by targeting TBC1D2 in cervical cancer (25), no significant changes were found between OC and peritumor tissues in our study (**Supplementary Figure S4A**). Then we focused on miR-373-3p and found that miR-373-3p negatively regulated TBC1D2 expression (**Figure 6B** and **Supplementary Figure S4B**). Further, we attempted to determine whether miR-373-3p could post-transcriptionally regulate TBC1D2 by luciferase reporter assay. 3’UTR regions of human TBC1D2 were cloned into pmirGLO dual-luciferase miRNA target expression vector and co-transfected with miR-373-3p mimic. The relative luciferase activity decreased significantly in TBC1D2-WT reporter compared with TBC1D2-MUT reporter, primarily because that miR-373-3p directly combined with the binding site on TBC1D2 3’UTR reporter (**Figure 6C** and **Supplementary Figure S4C**). Furthermore, we confirmed that miR-373-3p inhibited the invasion ability of OVCAR3 cells through downregulating TBC1D2 (**Figures 6D–G**). Additionally, we demonstrated that miR-373-3p could regulate the expression of E-cadherin and N-cadherin by modulating TBC1D2 expression (**Figure 6H**). These findings suggested that TBC1D2 played a critical role in OC cell invasion as a target of miR-373-3p.

## Discussion

Metastasis is definitely responsible for limited treatment and high mortality of epithelial ovarian cancer (26). Understanding the underlying mechanism of ovarian cancer metastasis at the molecular level will provide new insights into developing potential therapeutic targets and improving treatment (27). TBC domain-containing RAB-specific GTPase-activating proteins (TBC/RABGAPs), as negative regulators of RABs, are required for precise coordination of cell budding, transport, cytokinesis, membrane trafficking, and vesicle fusion (5). Recently, some TBC/RABGAPs were demonstrated as oncoproteins, which regulated cellular events relevant for oncogenesis and metastasis. For instance, Christine et al. found that TBC domain protein USP6/TRE17 could regulate HeLa cell migration and cytokinesis (28). Qi TF et al. demonstrated that elevated TBC1D7 expression promoted the invasion of melanoma cells *in vitro*, partly by modulating the activities of secreted matrix metalloproteinases 2 and 9 (29). In addition, Chen et al. reported that TBC1D8 drove oncogenesis and metabolic reprogramming of aggressive ovarian cancer cells (30). However, the clinical significance and potential roles of TBC1D2 were rarely explored in cancers.

This study investigated the expression profile of TBC1D2 in ovarian cancer and its clinical implications. We observed that high mRNA expression of TBC1D2 was strikingly associated with poor prognosis of OC patients. We attempted to evaluate the prognostic value of TBC1D2 in OC and our results indicated that it may be a promising prognostic biomarker for OC. Interestingly, previous studies of different cancers involving molecules in TBC1 domain family drew similar conclusions. For instance, being associated with poor outcome in breast carcinoma, TBC1D24 promoted cell proliferation through IGF1R/PI3K/AKT pathway (31). Being significantly up-regulated in OC cells and tissues, TBC1D8 drove OC development and metabolic reprogramming, and serves as an independent prognostic factor for OC patients (30). Meanwhile, we concluded in this study that high TBC1D2 expression negatively affects the prognosis of OC patients.

Aggravated cell invasion and migration lead to tumour metastasis, which is a hallmark of cancer and a non-negligible cause of cancer-related death, particularly in ovarian cancer (3, 32). Our data demonstrated that high TBC1D2 levels contributed to OC cell proliferation, invasion, and migration. In addition, different molecular biological functions of TBC1D2 have been explored in several studies. Bernadette et al. found that TBC1D2 coordinated Rac1 with Rab7 during autophagy in normal human keratinocytes. TBC1D2 overexpression induced the accumulation of enlarged autophagosomes, whereas TBC1D2 depletion significantly delayed autophagic flux (8). In addition, originally being an immunogenic tumour antigen, TBC1D2 may play a role in regulating cancer cell differentiation and growth (33). In conclusion, it could be suggested that TBC1D2 plays a vital role as an oncogene and promotes OC development.

**Figure 7:**
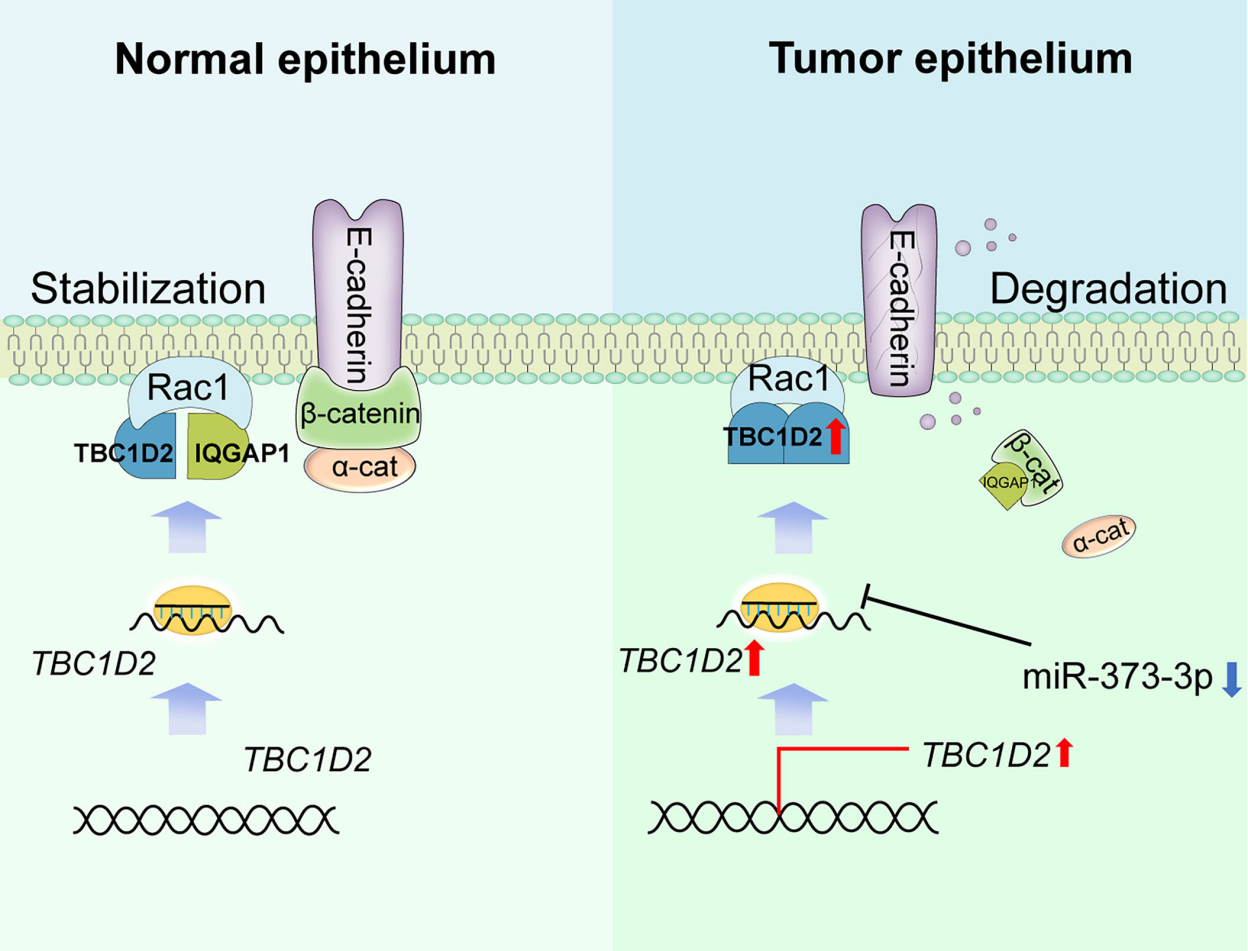
Summary of study. Schematic depicting the underlying mechanisms through which TBC1D2, negative regulated by miR-373-3p, promotes ovarian cancer metastasis *via* E-cadherin degradation induced by disintegration of Rac1-IQGAP1 complex.

Frasa MA et al. demonstrated that TBC1D2 promotes E-cadherin degradation *via* lysosomes (7). Similarly, we found that TBC1D2 played a vital biological role mainly in regulation of E-cadherin-mediated intercellular adhesion *via* modulating Rac1-IQGAP1 complex. To be more specific, Rac1-IQGAP1 complex and E-cadherin-mediated intercellular adhesion were stabilized when TBC1D2 was at a low expression level in normal ovarian epithelial cell. However, TBC1D2 was apt to competitively bind to Rac1 when TBC1D2 was at a relatively high expression level in OC epithelial cell. Subsequently, Rac1-IQGAP1 complex disintegrated and the free IQGAP1 was apt to competitively bind to β-catenin which was located in the adherens junctions, leading to dissociation of the intercellular adhesion and lysosomal degradation of E-cadherin, finally promoted cell invasion (**Figure 7**). Marei H et al (34). drew a similar conclusion that increased Rac1-IQGAP1 binding leading to reduced cell migration *via* stabilizing cadherin-mediated intercellular adhesions.

In addition, tumour immune infiltration is critical for OC progression. The current results suggested that TBC1D2 overexpression facilitates the infiltration of B cells, myeloid dendritic cells, and macrophages. To the best of our knowledge, immune cells infiltrating tumour microenvironment secrete interleukin-6 (IL-6), tumour necrosis factor-alpha (TNFα), transforming growth factor-beta (TGFβ), and growth factors to generate feedback loops that support tumour progression and metastasis (35–37). From this perspective, we can also conclude that high TBC1D2 expression levels probably promote OC metastasis. Furthermore, these findings indicate that TBC1D2 may be a promising target of OC metastasis and effectively response to immune therapy in OC management.

miRNAs are well known to play essential roles in regulating tumour development, particularly *via* RNA silencing or post-transcriptional regulation (20). Serva A et al. stated that TBC1D2 was identified as a miR-17-5p target gene in HeLa cells, and TBC1D2 depletion caused a decrease in intracellular transferrin (25). Interestingly, we predicted a new candidate microRNA targeting TBC1D2 based on three online databases. Additionally, we speculated that miR-373-3p binds directly to 3’UTR of TBC1D2 and induces mRNA degradation. miR-373 belongs to miR-371-3 cluster, which is transcribed from a location on chromosome 19q13.4 (38). Several studies confirmed the potential carcinogenic role of miR-373-3p in breast cancer (39), colorectal cancer (40) and testicular germ cell tumour (38). Surprisingly, this study revealed that miR-373-3p could promote OC cell invasion by downregulating TBC1D2 expression. Besides, numbers of studies on miR-373-3p involving liver cancer (41), gliomas (42) and gastric cancer (43) were also consistent with our findings that miR-373 has a potential anti-cancer effect.

In summary, our findings demonstrate that TBC1D2 serves as an oncogene in ovarian cancer and high expression level of TBC1D2 is associated with poor prognosis. In particular, TBC1D2 probably promotes OC metastasis *via* lysosomal degradation of E-cadherin induced by disintegration of Rac1-IQGAP1 complex. These findings suggest that TBC1D2 may be a promising and therapeutic target for treating OC.

## Data Availability Statement

The datasets presented in this study can be found in online repositories. The names of the repository/repositories and accession number(s) can be found in the article/**Supplementary Material**.

## Ethics Statement

The studies involving human participants were reviewed and approved by The Ethics Committee of the first hospital of Lanzhou university. The patients/participants provided their written informed consent to participate in this study. The animal study was reviewed and approved by the Animal Care Committee of the FMMU.

## Author Contributions

All authors of this paper have directly participated in the planning, execution, or analysis of the study. JimT, XLia, and DWa were involved in the acquisition, analysis, and interpretation of the data and drafting of the manuscript. JinT, TL, and HL were involved in tissue sample collection. ZY, DWu, and XLiu were involved in the analysis and interpretation of data. SL and YY were involved in the conception and design of the study, and critically revising the manuscript and overall study supervision. All authors read and approved the final manuscript.

## Funding

This study was supported by the National Natural Science Foundation of China (No. 81960278), the Outstanding Youth Funds of Science and Technology Department of Gansu Province (No. 20JR5RA371), the Natural Science Foundation of Gansu Province (No. 20JR10RA693) and the Foundation of the First Hospital of Lanzhou University (no. LDYYLL2022-87).

## Conflict of Interest

The authors declare that the research was conducted in the absence of any commercial or financial relationships that could be construed as a potential conflict of interest.

## Publisher’s Note

All claims expressed in this article are solely those of the authors and do not necessarily represent those of their affiliated organizations, or those of the publisher, the editors and the reviewers. Any product that may be evaluated in this article, or claim that may be made by its manufacturer, is not guaranteed or endorsed by the publisher.

## Abbreviations

OC, ovarian cancer; KEGG, Kyoto Encyclopedia of Genes and Genomes; GO, Gene oncology; qRT-PCR, Quantitative Real-Time Polymerase Chain Reaction; IHC, Immunohistochemistry; EdU, 5-Ethynyl-2’-deoxyuridine; TBC1D2, TBC1 Domain Family Member 2; RAC1, Rac Family Small GTPase 1; IQGAP1, IQ Motif Containing GTPase Activating Protein 1; OS, overall survival; WB, western blotting.
