# Supplementary material for: TBC1D2 Promotes Ovarian Cancer Metastasis via Inducing E-Cadherin Degradation
Source: Front Oncol. 2022 Apr 27;12:766077. doi: 10.3389/fonc.2022.766077 (PMC9091366; doi:10.3389/fonc.2022.766077)
Supplement: Supplementary file 1 [file DataSheet_1.docx]

Supplementary Material

# Supplementary Figures and Tables

## Supplementary Figures


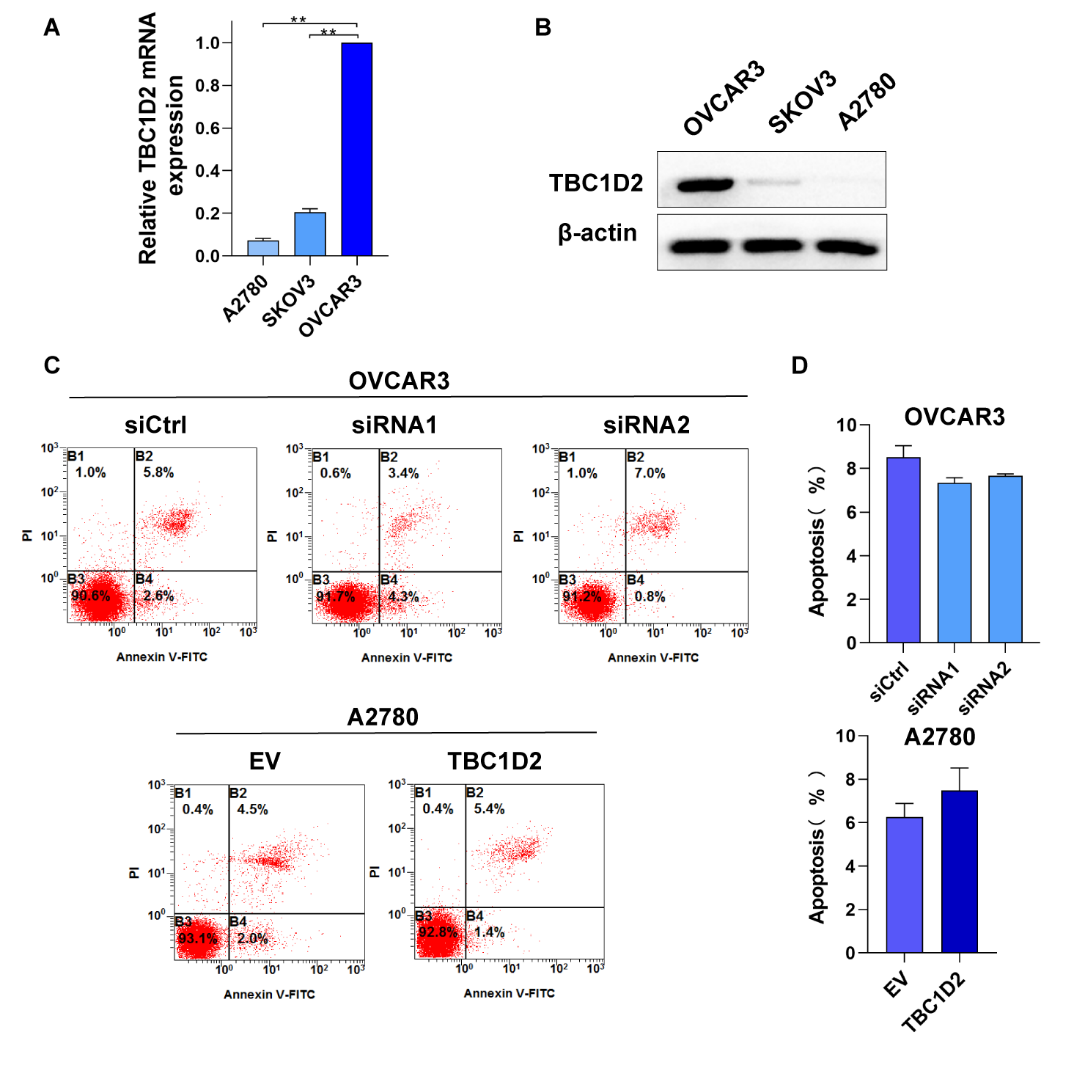


**Supplementary Figure 1.** Cell lines screening and apoptosis assays.

(A-B) qRT-PCR and Western blotting were performed to detect the expression of TBC1D2 in three ovarian cancer cell lines.

(C-D) Apoptosis indicated by Annexin V and PI staining in OVCAR3 and A2780 cells treated as indicated was analyzed by flow cytometry. siRNA1 or siRNA2, siRNA against TBC1D2; siCtrl, control siRNA; EV, empty vector; OE, overexpression vector encoding TBC1D2.


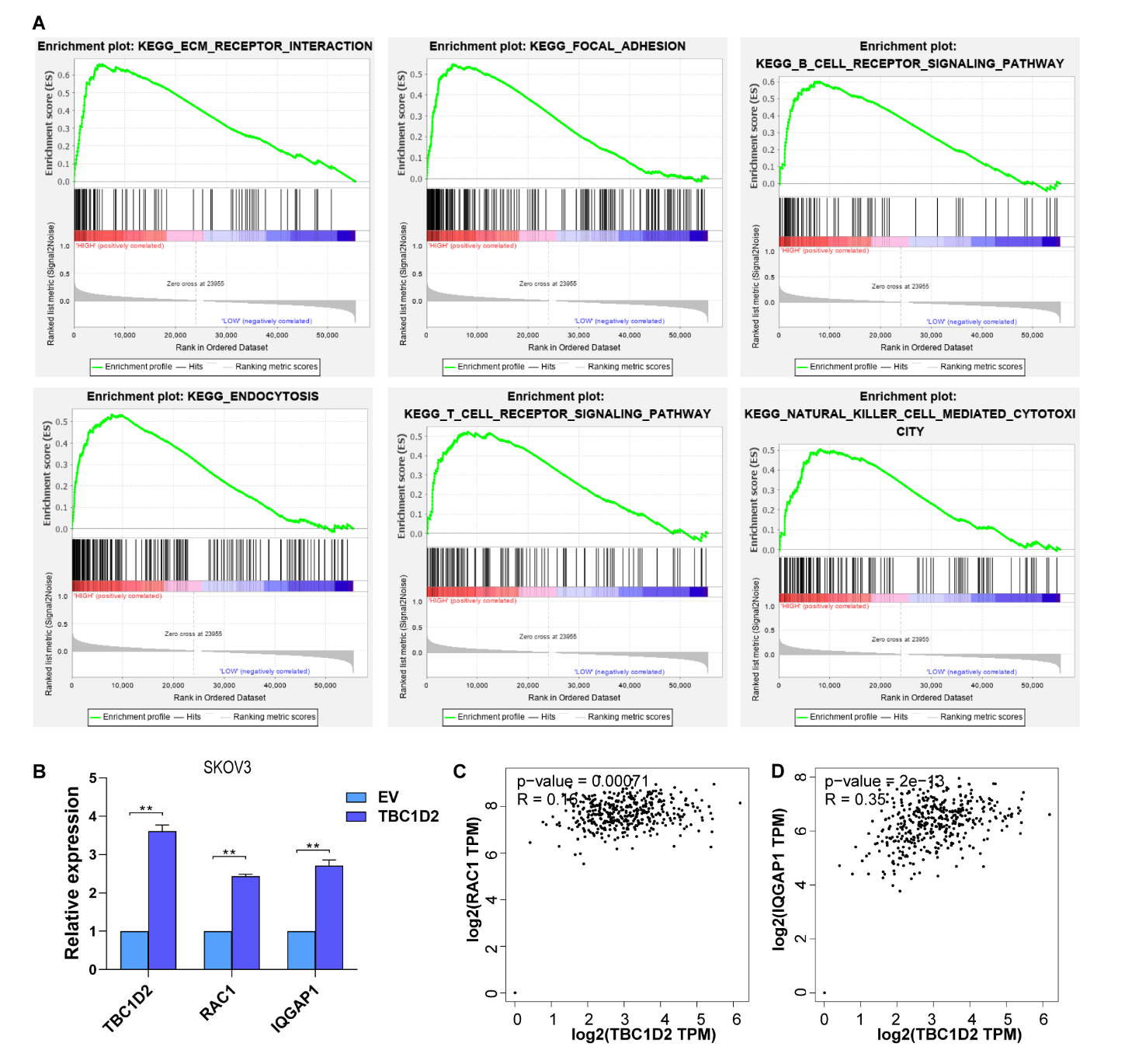


**Supplementary Figure 2.** GSEA and correlation analyses.

(A) Gene set enrichment analysis was performed by GSEA software.

(B) qRT-PCR analysis of TBC1D2, RAC1 and IQGAP1 in SKOV3 cells treated as indicated.

(C-D) The correlation between RAC1 or IQGAP1 and TBC1D2 was analyzed on GEPIA2 website.


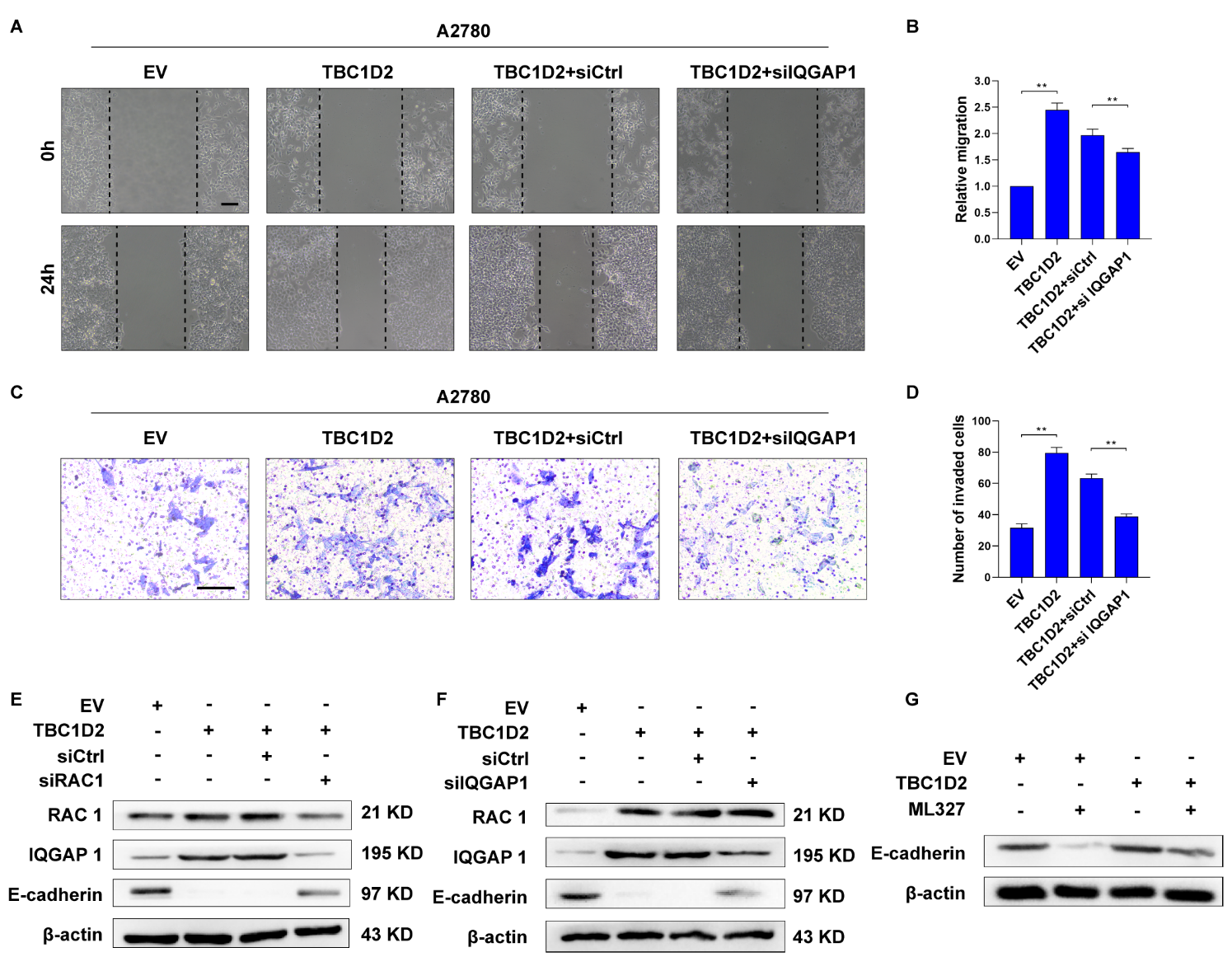


**Supplementary Figure 3.** TBC1D2 promotes cell invasion by decreasing E-cadherin via disassembling Rac1-IQGAP complex.

(A-D) Representative images of wound healing assay and transwell invasion assay in A2780 cells treated as indicated. EV, empty vector; TBC1D2, overexpression vector encoding TBC1D2; siCtrl, control siRNA; siIQGAP1, siRNA against IQGAP1.

(E-F) Western blotting images of RAC1, IQGAP1, E-cadherin and β-actin in A2780 cells treated as indicated.

(G) Western blotting of E-cadherin and β-actin in A2780 cells treated as indicated.


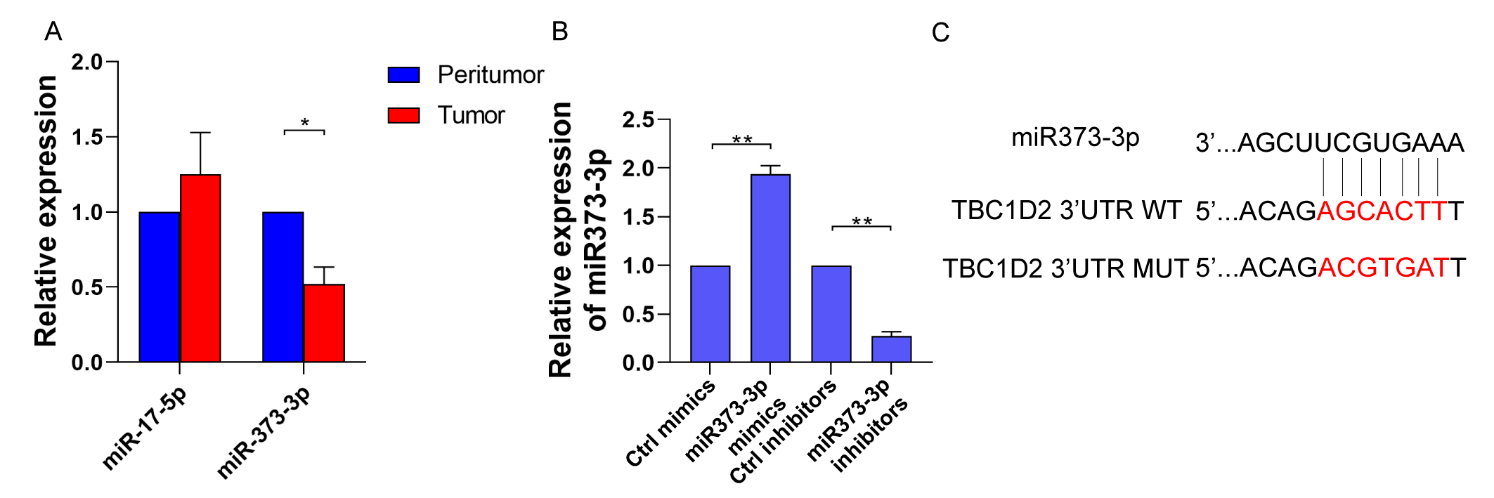


**Supplementary Figure 4.** miRNA expression in ovarian cancer and the UTR vector design.

(A) qRT-PCR of miR-17-5p and miR-373-3p in ovarian cancer and peritumor tissue.

(B) qRT-PCR was used to validate the expression of miR-373-3p mimic and inhibitor.

(C) miR-373-3p targeted seed sequences, wild type and mutational type of TBC1D2 3’UTR were designed.
